# Tracheotomy for Diphtheritic Croup

**Published:** 1880-05

**Authors:** 


TRACHEOTOMY FOR DIPHTHERITIC CROUP.

DR. FITZAN (*Berlin. Klin. Wochenschrift*, 1879), within two years, has performed tracheotomy for diphtheritic croup in thirteen cases, with twelve cures. One of these was in a child, only twenty months old. The patient who died was operated upon in extremis and died during the operation. The extremely favorable result is ascribed by the Doctor to the following precautions: First, that both, before and after the operation, he absolutely avoided all depressing therapeutics, and used tonics and strengthening remedies; second, that he caused the patients to diligently inhale, through throat and canula, a one-half per cent.

solution of salicylic acid of a temperature of 28 to 30 ° R. He continues these inhalations after the operation in even the most hopeless cases. He does not operate in septic-gangrenous cases. —*Am. Journal of Obstetrics.*

